# Purification and Characterization of Glutaminase Free Asparaginase from *Enterobacter cloacae*: *In-Vitro* Evaluation of Cytotoxic Potential against Human Myeloid Leukemia HL-60 Cells

**DOI:** 10.1371/journal.pone.0148877

**Published:** 2016-02-18

**Authors:** Islam Husain, Anjana Sharma, Suresh Kumar, Fayaz Malik

**Affiliations:** 1 Bacteriology Laboratory, Department of P. G. Studies and Research in Biological Science, Rani Durgavati University, Jabalpur, Madhya Pradesh, India; 2 Cancer Pharmacology Division, Indian Institute of Integrative Medicine, Jammu, India; Boston University Goldman School of Dental Medicine, UNITED STATES

## Abstract

Asparaginase is an important antileukemic agent extensively used worldwide but the intrinsic glutaminase activity of this enzymatic drug is responsible for serious life threatening side effects. Hence, glutaminase free asparaginase is much needed for upgradation of therapeutic index of asparaginase therapy. In the present study, glutaminase free asparaginase produced from *Enterobacter cloacae* was purified to apparent homogeneity. The purified enzyme was found to be homodimer of approximately 106 kDa with monomeric size of approximately 52 kDa and p*I* 4.5. Purified enzyme showed optimum activity between pH 7–8 and temperature 35–40°C, which is close to the internal environment of human body. Monovalent cations such as Na^+^ and K^+^ enhanced asparaginase activity whereas divalent and trivalent cations, Ca^2+^, Mg^2+^, Zn^2+^, Mn^2+^, and Fe^3+^ inhibited the enzyme activity. Kinetic parameters *K*_*m*_, *V*_*max*_ and *K*_*cat*_ of purified enzyme were found to be 1.58×10^−3^ M, 2.22 IU μg^-1^ and 5.3 × 10^4^ S^-1^, respectively. Purified enzyme showed prolonged *in vitro* serum (T_1/2_ = ~ 39 h) and trypsin (T_1/2_ = ~ 32 min) half life, which is therapeutically remarkable feature. The cytotoxic activity of enzyme was examined against a panel of human cancer cell lines, HL-60, MOLT-4, MDA-MB-231 and T47D, and highest cytotoxicity observed against HL-60 cells (IC_50_ ~ 3.1 IU ml^-1^), which was comparable to commercial asparaginase. Cell and nuclear morphological studies of HL-60 cells showed that on treatment with purified asparaginase symptoms of apoptosis were increased in dose dependent manner. Cell cycle progression analysis indicates that enzyme induces apoptosis by cell cycle arrest in G0/G1 phase. Mitochondrial membrane potential loss showed that enzyme also triggers the mitochondrial pathway of apoptosis. Furthermore, the enzyme was found to be nontoxic for human noncancerous cells FR-2 and nonhemolytic for human erythrocytes.

## Introduction

The use of enzymes to deprive neoplasm of essential nutrients offers a promising approach for treatment of tumor malignancies; asparaginase is cornerstone of them. Bacterial asparaginase (L-Asparaginase amidohydrolase, E.C. 3.5.1.1) is a selective and highly effective chemotherapeutic agent extensively used in first-line treatment of acute lymphoblastic leukemia (ALL), acute myeloblastic leukemia (AML) and other tumor malignancies in human [1]. The anti-neoplastic action of asparaginase is explained on the fact that certain tumor cells, more specifically lymphatic malignant cells are deficient in their ability to synthesize the non-essential amino acid asparagine *de-novo* due to absence of asparagine synthetase [2] but they require huge amount of asparagine to keep up their rapid malignant growth. To fulfill their nutritional requirement they use serum and cerebrospinal fluid (CSF) asparagine. The administration of asparaginase as a chemotherapeutic drug rapidly hydrolyses serum as well as CSF asparagine into aspartate and ammonia [3]. The nutritional stress induced by asparaginase by depletion of serum and CSF asparagine leads to DNA, RNA and protein biosynthesis inhibition in ALL, AML and other asparagine dependent tumor cells, resulting in subsequent apoptosis due to cell cycle arrest in G0/G1 phase [4]. However, normal cells remain unaffected due to presence of asparagine synthetase [5].

Since, 1961 anticancer activity of *Escherichia coli* asparaginase demonstrated by Broome [6], a wide variety of microorganisms were reported as asparaginase producers but still enzyme purified from *Escherichia coli* and *Erwinia chrysanthemi* has been used for clinical purposes [7]. Unfortunately, asparaginases obtained from both these organisms have several limitations including intrinsic glutaminase activity [8], shorter serum half life [9], low trypsin tolerance [10], mild hemolysis [11] and formation of anti-asparaginase antibodies [12]. These limitations led to cessation of therapeutic index of asparaginase therapy. Therefore, to get maximum therapeutic benefits, the search of glutaminase free asparaginase with effective chemotherapeutic potential is urgently required. In order to overcome some of the limitations of currently used asparaginases, previously we reported isolation of glutaminase free asparaginase producing indigenous bacterial strains [13] and fermentation process parameters were optimized for maximum yield of asparaginase [14]. In the current study, we have investigated purification and characterization of glutaminase free asparaginase from *E*. *cloacae*. Further, the cytotoxic activity of purified enzyme was investigated against panel of human cancer cell lines, MOLT-4, HL-60, MDA-MB-231, T47D, and non-cancerous breast epithelial (FR-2) cell line. In addition to this, the cytotoxicity of enzyme was validated against HL-60 cells by using various cellular and subcellular assays.

## Materials and Methods

### Ethics Statement

Blood samples were voluntarily drawn from Mr. Islam Husain (first Author) for these experiments. The blood samples were aseptically drawn from Mr. Husain by a trained technician at the Chandraker Cytodiagnostics and Pathology Centre, Jabalpur, Madhya Pradesh and ethical committee approval is not necessary for obtaining human blood for research purposes.

### Reagents

Standard molecular weight marker proteins were obtained from Bangalore Genei, India. Asparaginase, Diethylaminoethyl (DEAE) cellulose, Sephadex G-100, coomassie brilliant blue R-250, RPMI-1640, fetal bovine serum (FBS), penicillin, streptomycin, 3-(4,5-dimethylthiazole-2-yl)-2, 5-diphenyltetrazolium bromide (MTT), dimethylsulphooxide (DMSO), 4’,6-Diamidino-2-phenylindole dihydrochloride (DAPI), propidium iodide (PI), rhodamine-123, DNase-free RNase, proteinase-K, phenylmethanesulfonyl fluoride (PMSF), eukaryotic protease inhibitor cocktail, acridine orange and ethidium bromide were procured from Sigma chemical Co., USA. IPG strips were purchased from Bio-Rad Lab., USA. All other chemicals were used of analytical grade and procured from Himedia, Mumbai, India.

### Source of Bacterial Strain and Enzyme Production

The glutaminase free asparaginase producing strain *E*. *cloacae* (NCBI accession no: KF607094) was obtained from Bacterial Germplasm Collection Centre (BGCC no: 2389) from Rani Durgavati University, Jabalpur (M.P.), India, which was previously isolated in our Laboratory [13]. The strain was maintained on Luria-Bertani (LB) agar slant (pH 7) and stored at 4°C. For enzyme production, optimized semi synthetic broth medium was used [14]. Seed inoculum was prepared by adding a loopfull of 24 h old pure culture into 20 ml of above mentioned medium and incubated overnight at 37°C in a rotary shaking incubator at 180 rpm. The 2% inoculum (A600 = 0.6–0.8) of this culture was inoculated in 50 ml of medium and incubated at 37°C for 24 h at 180 rpm. Culture was harvested at 10,000 rpm and supernatant was used as crude enzyme.

### Asparaginase and Glutaminase Assays

The asparaginase activity was measured as described by Wriston [15], using Nesslerization reaction. Glutaminase activity of asparaginase was determined by Nessler’s method as described by Imada et al. [16]. One asparaginase unit (IU) is defined as the amount of enzyme that liberates 1μmol of ammonia min^-1^ under standard assay conditions. Protein concentration was determined according to the method of Lowry et al. [17], using bovine serum albumin (BSA) as standard. Specific activity of asparaginase is expressed as U mg^-1^ protein.

### Purification and Quantification of Asparaginase

Unless otherwise indicated, all the purification steps were performed at 4°C and chromatographic runs were monitored for protein at 280 nm. Asparaginase produced by *E*. *cloacae*, fractionated by adding powdered ammonium sulphate into clear supernatant. The maximum asparaginase activity was observed with the fraction precipitated at 60–90% saturation. The precipitate was collected by centrifugation at 10,000 rpm for 30 min and dissolved in minimum volume of 0.05 M Tris-HCl buffer, pH 8.6. The enzyme solution was dialyzed for 24 h against the same buffer. After that, the dialysate was loaded on diethylaminoethyl (DEAE) cellulose column (2×15 cm^2^, Sigma), pre-equilibrated with 0.05 M Tris-HCl buffer, pH 9.6 at a flow rate of 1 ml min^-1^. The absorbed protein was eluted with a linear gradient of KCl (0–200 mM), prepared in 0.05 M Tris-HCl buffer, pH 8.6. The fractions containing asparaginase activity were pooled, dialyzed against 0.05 M Tris-HCl buffer (pH 8.6). Further, the dialyzed sample from DEAE-cellulose column was loaded on pre-equilibrated Sephadex G-100 column (2×22 cm^2^, Sigma), with 0.05M Tris-HCl buffer, pH 8.6. The protein elution was done with the same buffer at a flow rate of 0.2 ml min^-1^, until no protein was seen in the eluate. All the active fractions were pooled, concentrated, dialyzed and used as purified enzyme.

### Determination of Molecular Weight and Homogeneity of Asparaginase by Electrophoresis

The subunit molecular weight of the purified asparaginase was estimated by SDS-PAGE under reducing conditions as described by Laemmli [18], using 12% separating gel (pH 8.8) and 5% stacking gel (pH 6.8). The native-PAGE of purified asparaginase was performed using 10% resolving gel (stacking gel was omitted) in Tris-glycine buffer (pH 8.5) at 4°C. The molecular weight of the purified asparaginase was determined in comparison with standard protein markers. Proteins were detected by staining with coomassie brilliant blue R-250.

### Two Dimension Gel Electrophoresis

Purified asparaginase was dissolved in rehydration buffer containing 7 M urea, 2 M thiourea, 4% CHAPS, 40 mM DTT (DL-dithiothreitol), and 1.0% IPG buffer (4–7). Before loading for 2-DE traces of bromophenol blue was added and centrifuged at 20,000 rpm for 10 min. A total of 300 μl of solubilization buffer containing 250 μg protein sample was incubated with the dry IPG (immobilized pH gradient) gel strips (pH 4–7 linear gradients 13 cm) at 20°C for 16 h. The first dimension separation was conducted at 20°C with an Ettan IPG phor system (GE Healthcare, USA). Three phase program was used for performing IEF as described by Gorg et al. [19]. Focused IPG strips were then equilibrated by first incubating them in an equilibration solution (6-M urea, 30% v/v glycerol, 2% w/v SDS, 50 mM Tris-HCl, pH 8.8) having 1% w/v DTT and a trace amount of bromophenol blue for 15 min, followed by 15 min incubation with 2.5% w/v iodoacetamide. The equilibrated IPG strip was placed on top of the SDS gel and overlaid with 5 ml of hot (45°C) agarose solution. The SDS gel cassette was inserted in the electrophoresis apparatus containing electrode buffer and run overnight (15 h) with a voltage setting of 50 V for 1 h and 100 V for 14 h at 4°C. After electrophoresis, the gel was stained with coomassie brilliant blue R-250. Molecular weight of the enzyme was measured according to the standard protein markers.

### Effect of pH and Temperature on Activity and Stability of Purified Asparaginase

The optimum pH for asparaginase activity was determined over a pH range of 4.5–10.5 using following buffer: 0.05 M potassium phosphate buffer, pH 4.5–7.5 and 0.05 M Tris-HCl buffer, pH 8.0–10.5. For pH stability, asparaginase was incubated with same buffers at 4°C for 24 h in the absence of substrate and enzyme activity was determined under standard assay. For determining the optimum temperature, enzyme was incubated at different temperatures (20–70°C) for 30 min and for thermal stability, enzyme was pre-incubated at desired temperature for 15 min and residual activity was determined.

### Effect of Various Effectors on Asparaginase Activity

Asparaginase activity was investigated in the presence of various metal ions, detergents and modulators [20–22] after 30 min of pre-incubation at 37°C, individually. Enzyme activity was determined under standard assay conditions and relative activity was expressed as the percentage ratio of the activity of the enzyme incubated with compound to that of the untreated enzyme.

### Substrate Specificity

Substrate specificity of purified asparaginase was determined with amide bond containing various substrates viz. L-asparagine, D-asparagine, DL-asparagine, L-glutamine, D-glutamine, L-glutamic acid, DL-aspartic acid, L-histidine, L-ornithine, BOC-L-asparagine, N-α-acetyl-L-asparagine, urea and acrylamide at the concentration of 0.01 M. Results were presented in the terms of percent relative activity.

### Kinetic Parameters of Purified Asparaginase

The Michaelis constant (*K*_*m*_), maximal velocity (*V*_*max*_) and turn over numbers (*K*_*cat*_) of purified asparaginase were determined using L-asparagine as substrate at the concentrations range of 0.2–24 mM in 0.05 M phosphate buffer (pH 7.5) at 37°C. The *K*_*m*_ and *V*_*max*_ were calculated from Lineweaver–Burk plots by using equation derived from non-linear regression analysis of curve. The K_cat_ value was calculated using the equation *K*_*cat*_ = *Vmax* / [E], where [E] is the enzyme concentration used in reaction. All the reactions were performed in triplicate and the average value was taken into consideration during all kinetic study [20].

### *In Vitro* Serum and Trypsin Half Life (T_1/2_)

In order to study the *in vitro* serum half life, purified asparaginase (50 IU, 0.5 ml volume) was mixed with 2.5 ml human serum, homogenized vigorously and sample was incubated at 37°C. Asparaginase activity was determined in 0.1 ml reactive plasma containing asparaginase at regular interval of 5 h till the enzyme was found to be active. To determine the trypsin tolerance (T_1/2_), 0.5 ml purified asparaginase (50 IU) was added to 2.5 ml of 0.05 M phosphate buffer (pH 7.5) containing 50 IU of trypsin [10]. Reaction mixture was homogenized vigorously, incubated at 37°C and the enzyme activity was measured at regular interval of 10 min.

### Antilymphoproliferative Activity of Purified Asparaginase

#### Cell cultures, growth conditions and treatment

Human T-lymphoblast acute lymphoblastic leukemia cell line MOLT-4, promyelocytic leukemia cell line HL-60, breast cancer cell lines MDA-MB-231 and T47D and non-cancerous breast epithelial cell line FR-2 were obtained from National Cancer Institute, Frederick, U.S.A. Cells were grown in RPMI-1640 medium containing 10% Fetal bovine serum (FBS), penicillin (100 U ml^-1^), and streptomycin (100 μg ml^-1^) in CO_2_ incubator (Thermocon Electron Corporation, USA) at 37°C with 98% humidity and 5% CO_2_ gas environment. Cells were treated with filter sterilized purified asparaginase solution during logarithmic growth phase.

#### Cytotoxicity assay

The cytotoxic effect of purified asparaginase was investigated by MTT assay [23]. Briefly, 1.2×10^4^ to 1.5×10^4^ exponentially growing cells were seeded in 96-well -plates, containing 200 μl RPMI-1640 medium, with 10% fetal bovine serum. Cells were treated with different concentrations of asparaginase. After 48 h, 10 μl/well of MTT dye (5 mg ml^-1^ stock) was added in each well and plates were incubated at 37°C in CO_2_ incubator for 4 h. The plates were centrifuged at 1600 rpm for 15 min and supernatant was discarded. The formazan blue crystal, formed by viable cells, was dissolved with 150 μl DMSO and the rate of color production was measured at 570 nm with ELISA reader. The O.D of control samples were considered to be 100% and accordingly the viability of other samples were calculated by using the following formula.

$$\%\;\mathrm{Viability}=\frac{\left(O.\mathrm{D Treated well}\right)}{\left(O.\mathrm{D Control well}\right)}\times 100$$

#### Assessment of cell morphology

Phase contrast microscopy was performed to assess the morphological changes on HL-60 cells after treatment with asparaginase. Approximately, 0.6x10^6^ cells were cultured in six-well plates and treated with different concentrations of asparaginase. Plates were incubated at 37°C in CO_2_ incubator with 5% CO_2_ for 24 h. After incubation, cells were subjected to photography. Apoptosis was assessed using a microscope (1×70, Olympus), and photographs were taken by using DP-12 camera.

#### DAPI staining of cells for nuclear morphology

In order to study the effect of asparaginase treatment on the level of apoptosis, HL-60 cells (0.6x10^6^) were cultured in six-well plates. Cells were treated with different concentrations of asparaginase and incubated at 37°C in CO_2_ incubator with 5% CO_2_ for 24 h. After incubation, cells were collected in flow tubes and centrifuged at 1600 rpm for 5 min at 4°C. Pellets were washed with PBS buffer and fixed in methanol for 30 min at 4°C. Cells were stained with 4’,6-diamidino-2-phenylindole dihydrochloride (DAPI) (1 μg ml^-1^) for 10 min, centrifuged and resuspended in 50 μl of mounting fluid (PBS:glycerol, 1:1). 10 μl of this cell suspension was spread on clean glass slide and covered with coverslip [24]. The slides were then observed for any nuclear morphological alterations and apoptotic bodies under an inverted fluorescence microscope (Olympus 1×70, magnification 30×) using UV excitation.

### Cell Cycle Progression Analysis

Distribution of cells in different phases of cell cycle was analyzed following treatment with asparaginase by flow cytometry. HL-60 cells (1×10^6^) were seeded in 6 well plate and treated with different concentrations of asparaginase. Plates were incubated at 37°C in CO_2_ incubator with 5% CO_2_ for 24 h. After incubation, cells were centrifuged at 1600 rpm and cell pellet was washed twice with ice-cold PBS, centrifuged and fixed with 70% ethanol and stored at 4°C overnight. After fixation, cells were further centrifuged and resuspended in 200 μl PBS and incubated with RNAse A at 37°C for 90 min. PBS was added to make its volume 500 μl and stained with propidium iodide (100 μg ml^-1^) for 30 min on ice in the dark, and then measured for DNA content using a BD FACS flow cytometer (Becton Dickinson, Franklin Lakes, NJ). Data were collected in list mode, and 10,000 events were analyzed for FL2-A vs FL2-W. Modfit software was used to distinguish different phases of the cell cycle [25].

### DNA Fragmentation Assay

The DNA fragmentation assay was performed as described earlier [26]. Briefly, cells of exponential growth phase were cultured in six well plates at a density of 2×10^6^ cells/2 ml and treated with different concentrations of asparaginase for 24 h. After treatment, cells were harvested by centrifugation at 1600 rpm for 5 min and washed with PBS containing 20 mM EDTA. The pellet was lysed in 250 μl of lysis buffer (100 mM NaCl, 5 mM EDTA, 10 mM Tris-HCl, pH 8.0 and 5% Triton X-100) containing 100 μg ml^-1^ of DNase-free RNase and incubated at 37°C for 90 min followed by 1 h incubation with proteinase-K (200 μg ml^-1^) at 50°C. The DNA was extracted with 150 μl of phenol for 1 min and centrifuged at 15,000 rpm for 2 min. The aqueous phase was further extracted with chloroform: isoamylalcohol (24:1) and centrifuged. DNA was precipitated from aqueous phase with three volumes of chilled alcohol and 0.3 M sodium acetate at -20°C overnight. The precipitate was centrifuged at 15,000 rpm for 10 min. The DNA pellet was washed with 80% alcohol, dried, dissolved in 50 μl TE buffer and electrophoresed in 1.8% agarose gel at 50 V for 1.5 h.

#### Acridine orange—Ethidium bromide (AO/EB) staining

For AO/EB staining, approximately 1×10^5^ cells ml^-1^ were cultured in six well plates and treated with different concentrations of asparaginase for 24 h. Cells grown in culture medium alone were used as control. After treatment, cells were harvested at 1600 rpm for 5 min, washed once with PBS and resuspended in AO/EB solution (100 mg ml^-1^; 1:1 ratio). Cells were immediately visualized under a fluorescence microscope (Olympus 1×70, magnification 30×), where apoptotic cells showed orange and live cells showed green nuclei. Quantification of apoptotic cells was performed as earlier described by Ribble et al. [27]. The percentage of apoptotic cells within the overall population of total cells was defined as apoptosis rate.

#### Measurement of mitochondrial membrane potential (MMP) loss

For measurement of mitochondrial membrane potential (MMP) loss, HL-60 cells (1×10^6^) were seeded in 12-well plates, treated with different concentrations of asparaginase and incubated at 37°C in CO_2_ incubator for 24 h. After treatment, Rhodamine-123 (200 nM ml^-1^) was added 30 min before termination of the experiment. Cells were collected and washed once with PBS and centrifuged. The cell pellet was resuspended into 500 μl PBS and mitochondrial membrane potential was measured in the FL-1 channel of the flow cytometer (Becton Dickinson, Franklin Lakes, NJ) [23].

### Toxicological Evaluation of Purified Asparaginase on Noncancerous Human Cell Line and Erythrocytes

#### Cytotoxicity assay

The cytotoxic effect of purified asparaginase on noncancerous human cell line FR-2 was investigated by MTT assay as described above.

#### Hemolysis assays

Hemolysis assay was performed to investigate the hemolytic effect of purified asparaginase on human blood (erythrocytes). The blood agar plate composed of 5% fresh defibrinated human blood was prepared. 25 μl crude and purified asparaginases were separately placed into the holes previously punched in the blood agar plate. After incubation at 37°C for 24 h, the plate was examined for transparent or translucent halo zone on it [28].

Quantitative hemolytic assay was performed according to the method of Bulmus et al. [29]. Briefly, defibrinated human blood (erythrocytes) were taken and washed three times with 150 mM NaCl and cells were suspended in 100 mM sodium phosphate buffer, pH 7.2. Cells were incubated at 37°C with different concentration of purified asparaginase for 24 h. After incubation, cells were centrifuged at 2500 rpm for 15 min and supernatant was read at 541 nm.

### Statistical Analysis

All experiments were performed in triplicate and data reported as mean ± SD. Statistical analysis was done by using student t-test and p value <0.05 was considered to be statistically significant in this study.

## Results

### Purification of Asparaginase

**Fig 1:**
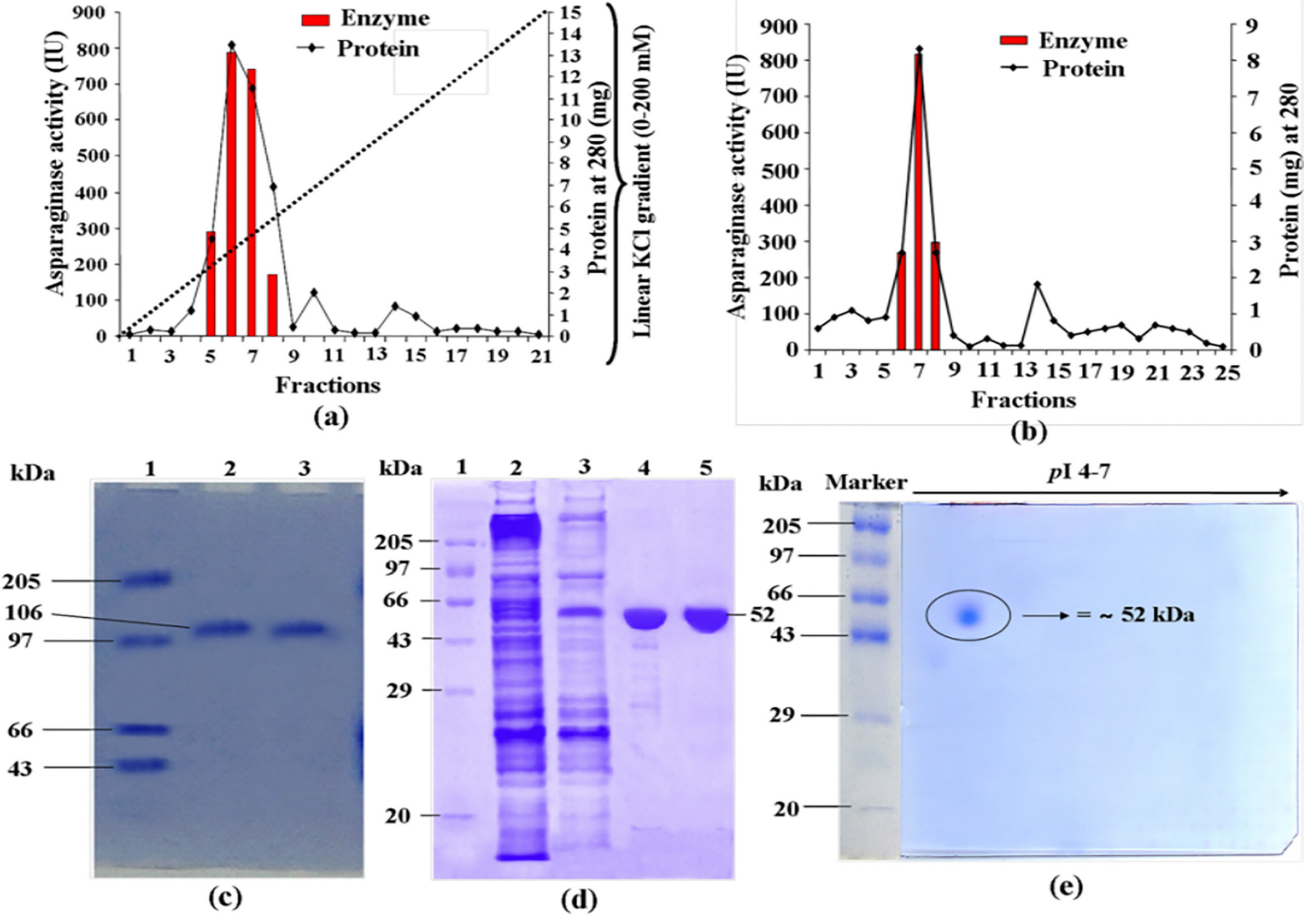
Purification of asparaginase from *E*. *cloacae*. Elution profiles of asparaginase **(a)** Cation exchange chromatography of 60–90% ammonium sulphate precipitated fraction on DEAE-cellulose column (2×15 cm^2^, Sigma). The column was pre-equilibrated with 0.05 M Tris-HCl buffer (pH 9.6) and absorbed protein was eluted with a linear gradient of KCl (0–200 mM) prepared in 0.05 M Tris-HCl buffer (pH 8.6). **(b)** Gel filtration of fractions (5,6,7 and 8) from DEAE-cellulose column on a Sephadex G-100 column (2×20 cm^2^, Sigma), which was pre-equilibrated with 0.05 M Tris-HCl buffer (pH 8.6) and eluted with the same buffer at a flow rate of 0.2 ml min^-1^. **(c)** Assessment of homogeneity and molecular weight analysis of purified asparaginase on Native-PAGE, **Lane 1**- Protein marker; **Lane 2** and **3-** Sephadex G-100 purified asparaginase. **(d)** SDS-PAGE, **Lane 1**- Protein marker; **Lane 2**- Cell free crude asparaginase; **Lane 3-** Ammonium sulphate precipitated asparaginase; **Lane 4**-DEAE-cellulose purified asparaginase and **Lane5-** Sephadex G-100 purified asparaginase. **(e)** Two-dimensional electrophoretic resolution of purified asparaginase. Purified enzyme (30 μg) after resolved by IEF and SDS-PAGE gel was visualized by coomassie brilliant blue staining. Molecular weight was calculated with standard molecular weight markers.

Glutaminase free asparaginase from *E*. *cloacae* was purified by the combination of several steps including ammonium sulphate precipitation, ion-exchange chromatography on DEAE-cellulose and gel filtration on Sephadex G-100 column. Total 4200 asparaginase units were stepwise precipitated by adding powdered ammonium sulphate with a saturation range of 30, 60, and 90% fraction. The maximum asparaginase units (2856 IU) were precipitated with a saturation range of 60–90%, yielding 523.36 mg proteins and specific activity was approximately 5.45 IU mg^-1^ protein. Dialysate was loaded on pre-equilibrated DEAE-cellulose column and bound proteins were eluted with 0–200 mM KCl. The fractions no. 5, 6, 7, and 8 having maximum asparaginase activity were eluted at approximately 45–75 mM KCl solution (Fig 1A). For further purification, active fractions were concentrated, dialyzed and loaded onto a pre-equilibrated Sephadex G-100 column. Highest asparaginase activity containing fractions no. 6, 7, and 8 (Fig 1B) were concentrated, stored at -40°C, and used as purified asparaginase. The purification results of asparaginase are summarized in Table 1. The specific activity yield of the purified enzyme was calculated to be 105.07 IU mg^-1^ protein, under optimal assay condition using asparagine as a substrate. The fold purification of enzyme was 119.39, with the final yield of 33% compared to the crude extract. Purified asparaginase did not exhibit intrinsic glutaminase activity.

**Table 1:** Summary of various steps involved in purification of asparaginase from *E*. *cloacae*.

Purification step	Total activity (IU)^a^	Total Protein (mg)	Specific activity (IUmg^-1^)	Fold Purification	Yield (%)
Crude extract	4200	4746	0.88	1	100
(NH_4_)_2_SO_4_ precipitation	2856	523.36	5.45	6.19	68
DEAE-Cellulose	1992	39.25	50.75	57.67	47.42
Sephadex G-100	1394	13.27	105.07	119.39	33

^a^One unit of asparaginase (IU) is defined as the amount of enzyme that librates 1 μmol of ammonia min^-1^ at 37°C.

### Determination of Molecular Weight and Isoelectric Point of Purified Asparaginase

To analyze the homogeneity and molecular weight of purified asparaginase, native-PAGE and SDS-PAGE were performed. As shown in Fig 1C, the molecular mass of the purified enzyme was estimated to be approximately 106 kDa as assessed by Native-PAGE. Further, single band by SDS-PAGE analysis showed the subunit molecular weight of approximately 52 kDa (Fig 1D). The SDS-PAGE analysis indicates that purified enzyme is a dimeric protein consisted of two identical molecular weight subunits, while native-PAGE exhibited single band indicating high purity of enzyme. Two-dimension polyacrylamide gel electrophoresis (2D-PAGE) was used to determine the isoelectric point (p*I*) of enzyme. The purified enzyme showed a band near 4.5 p*I* point of 4–7 nonlinear gradients IPG strip as well as 2D-PAGE gel (Fig 1E).

### Effect of pH and Temperature on Enzyme Activity and Stability

**Fig 2:**
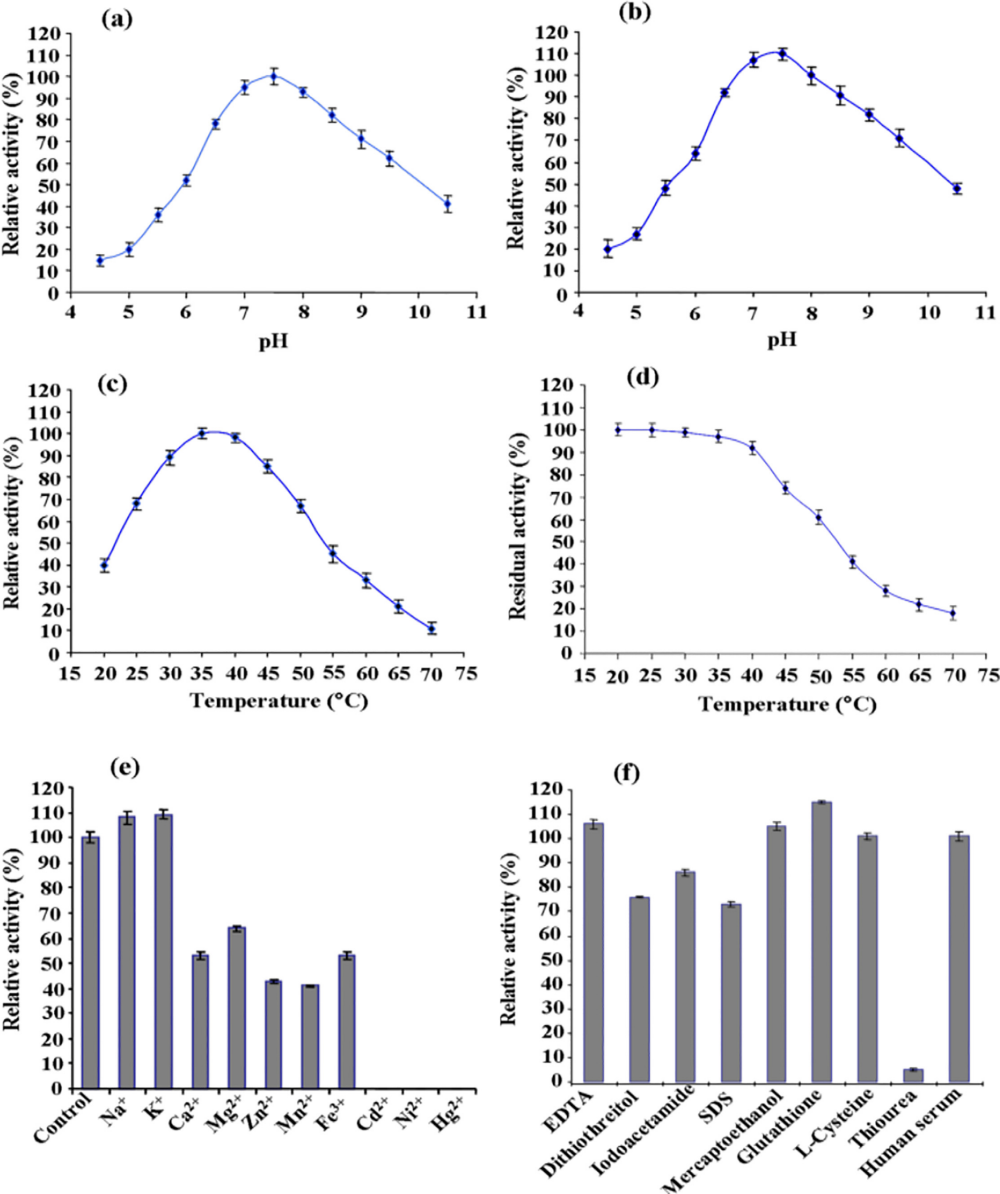
Biochemical properties of asparaginase purified from *E*. *cloacae*. Optimum pH **(a)** and pH stability of asparaginase **(b)** were determined by assessing the enzyme activity in the pH range of 4.5–10.5 with various pH buffers as follows: pH 4.5–7.5, 50 mM Potassium phosphate buffer; pH 8.0–10.5, 50 mM Tris-HCl buffer. Optimum temperature on the activity **(c)** and the thermostability of asparaginase **(d)** were determined at various temperatures (20–70°C). Effect of various metal ions **(e)** and modulators **(f)** on the asparaginase activity. Asparaginase was mixed with the corresponding metal salts (NaCl (50 mM), (KCl (150 mM), (CaCl_2_ (150 mM)_,_ (MgCl_2_ (40 mM)_,_ (ZnCl_2_ (100 mM), (MnCl_2_ (100 mM), (FeCl_3_ (100 mM), (CdCl_2_ (10 mM), (NiCl_2_ (10 mM), (HgCl_2_ (100 mM) or modulators EDTA (5 mM), DDT (5 mM), iodoacetamide (5 mM), SDS (2 mM), 2-mercaptoethanol (0.5 mM), glutathione (0.5 mM), L-cysteine (25 mM), thiourea (1 mM), and human serum (10%) in 50 mM phosphate buffer (pH 7.5) for 30 min at 37°C and enzyme activity was measured by Nesslerization reaction. No addition was used as control. Each value represents the mean ±SD for three determinations.

The effect of pH on enzyme activity and stability was monitored from pH 4.5–10.5 and temperatures in a range 20–70°C, using asparagine as a substrate. Purified enzyme was found to be active and stable at pH range from 6 to 9 with the optimum pH 7–8 (Fig 2A and 2B). As shown in Fig 2C, the enzyme was more active at 25–45°C, exhibiting maximal activity at temperature 35–40°C, whereas the enzyme activity decreased rapidly after 45°C. The enzyme was stable and retained over 90% of its initial activity upto the temperature 40°C, but enzyme activity lost quickly when enzyme was incubated above 40°C (Fig 2D).

### Effect of Various Effectors and Modulators on Asparaginase Activity

The effect of various effectors on enzyme activity is summarized in Fig 2E and 2F. Under incubation condition, Na^+^ and K^+^ ions acted as enhancers whereas Ca^2+^, Mg^2+^, Zn^2+^, Mn^2+^, and Fe^3+^ were found as detrimental to enzyme activity. However, asparaginase activity did not detect when enzyme was incubated with metal ions viz. Cd^2+^, Ni^2+^ and Hg^2+^. Reducing agents 2-mercaptoethanol and glutathione (reduced), amino acid L-cysteine and metal chelator, EDTA slightly stimulated enzyme activity while, dithiothreitol, iodoacetamide inhibited enzyme activity. Asparaginase activity almost diminishes when enzyme was incubated with thiourea.

### Substrate Specificity of Purified Asparaginase

Purified enzyme showed highest activity when L-asparagine used as a substrate and no activity was observed when L-glutamine, D-glutamine, L-glutamic acid, BOC-L-asparagine, N-α-acetyl-L-asparagine, urea and acrylamide were used separately as a substrate in place of L-asparagine (Table 2).

**Table 2:** Substrate specificity of purified asparaginase from *E*. *cloacae*.

Substrate	Concentration (mM)	Relative activity (%)^a^
L-Asparagine	10	100±1.9
D-Asparagine	10	2±0.4
DL-Asparagine	10	4±0.3
L-Glutamine	10	N.D.
D-Glutamine	10	N.D.
L-Glutamic acid	10	N.D.
DL-Aspartic acid	10	1.3±0.4
L-Histidine	10	1.1±0.3
L-Ornithine	10	1.2±0.2
BOC-L-Asparagine	10	N.D.
N-α-Acetyl-L-Asparagine	10	N.D.
Urea	10	N.D.
Acrylamide	10	N.D.

N.D. = Not detected

^a^Relative activity shown in the table. Each value represents the mean ±SD for three determinations.

### Kinetic Parameters

**Fig 3:**
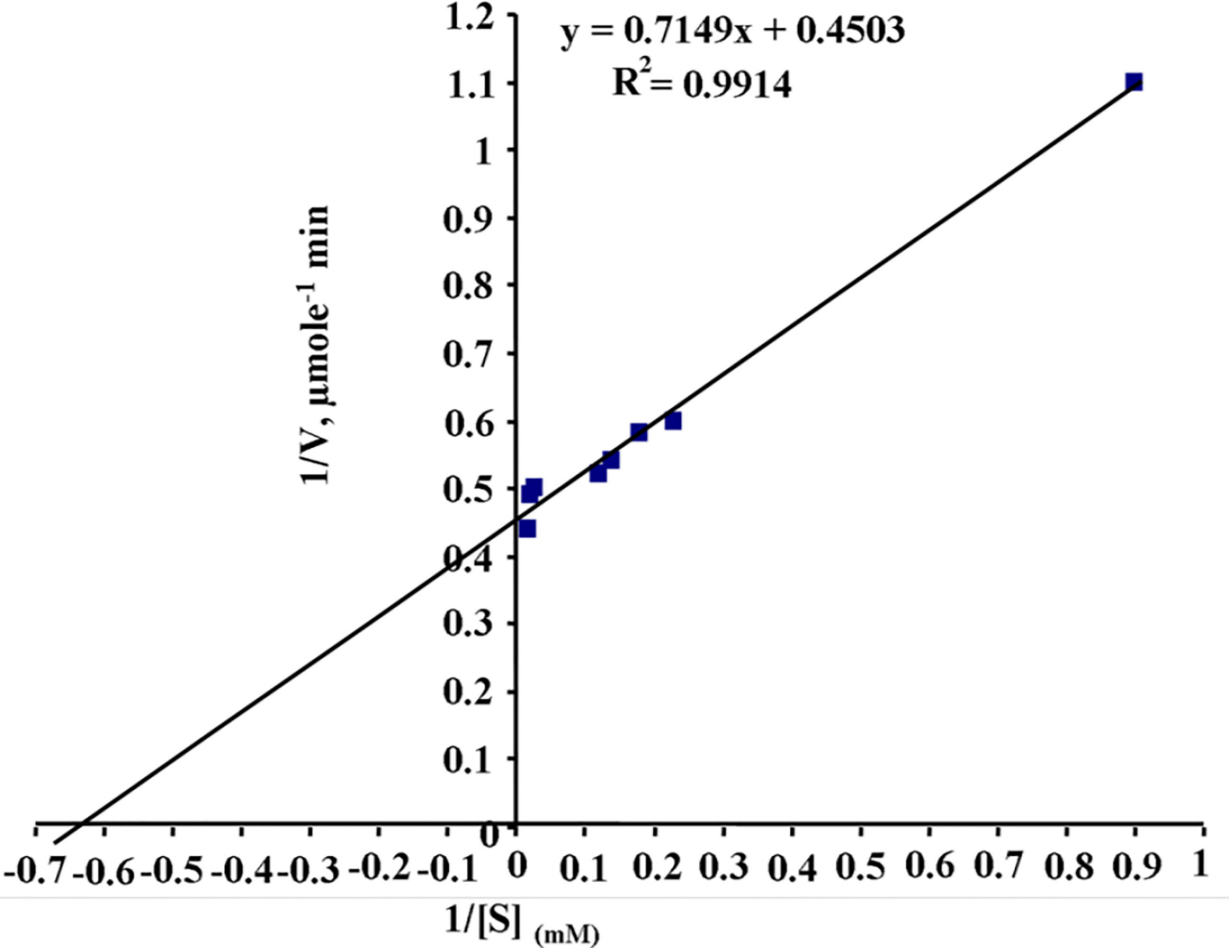
The Lineweaver-Burk plot of *E*. *cloacae* asparaginase for calculating kinetic parameters. The concentrations of the substrate L-asparagine were between 0.2–24 mM. The *K*_*m*_, *V*_*max*_ and *K*_*cat*_ of purified asparaginase were 1.58×10^−3^ M, 2.22 IU μg^-1^, and 5.3×10^4^ S^-1^, respectively. All data were average value of triplicate measurement.

According to the results that are summarized in Fig 3, the Michaelis constant (*K*_*m*_) and maximal velocity (*V*_*max*_) of purified asparaginase from *E*. *cloacae* were 1.58×10^−3^ M and 2.22 IU μg^-1^, respectively. The turnover numbers (*K*_*cat*_) of the purified enzyme was found to be 5.3 × 10^4^ S^-1^.

### *In Vitro* Serum and Trypsin Half Life

**Fig 4:**
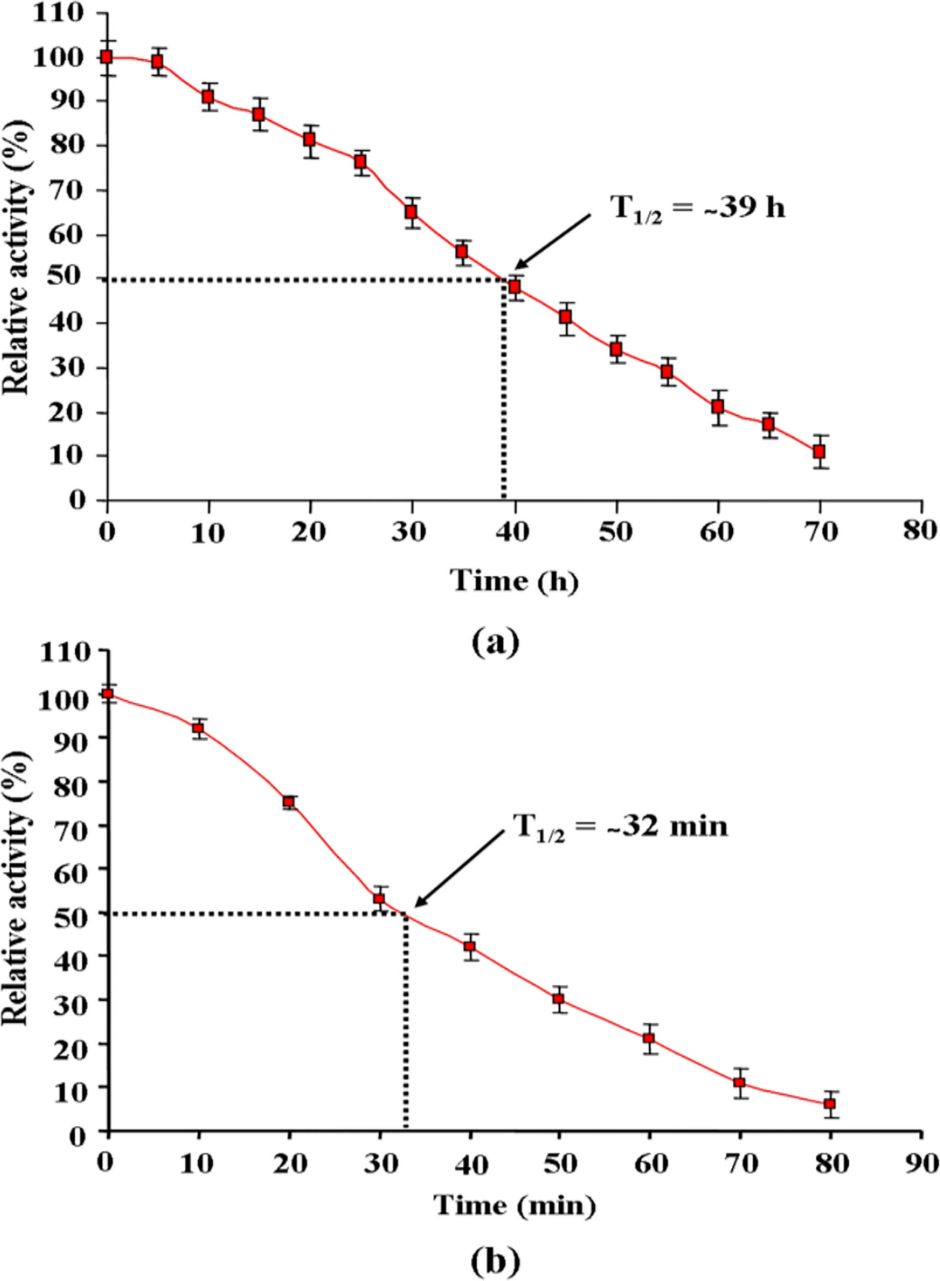
Physiological properties of asparaginase purified from *E*. *cloacae*. *In vitro* **(a)** serum and **(b)** trypsin half life. Asparaginase was incubated with serum and trypsin, separately and relative activity was measured on the basis of the highest activity of purified asparaginase as 100%. Each value represents the mean ±SD for three determinations.

The *in vitro* half life of *E*. *cloacae* asparaginase in human blood serum is illustrated in Fig 4A. The results showed a reduction of 50% initial activity after approximately 39 h of incubation. Results of trypsin tolerance are illustrated in Fig 4B, the purified enzyme exhibited a reduction of 50% of initial activity after approximately 32 min on incubation at 37°C.

### Asparaginase Reduced Cell Viability

**Fig 5:**
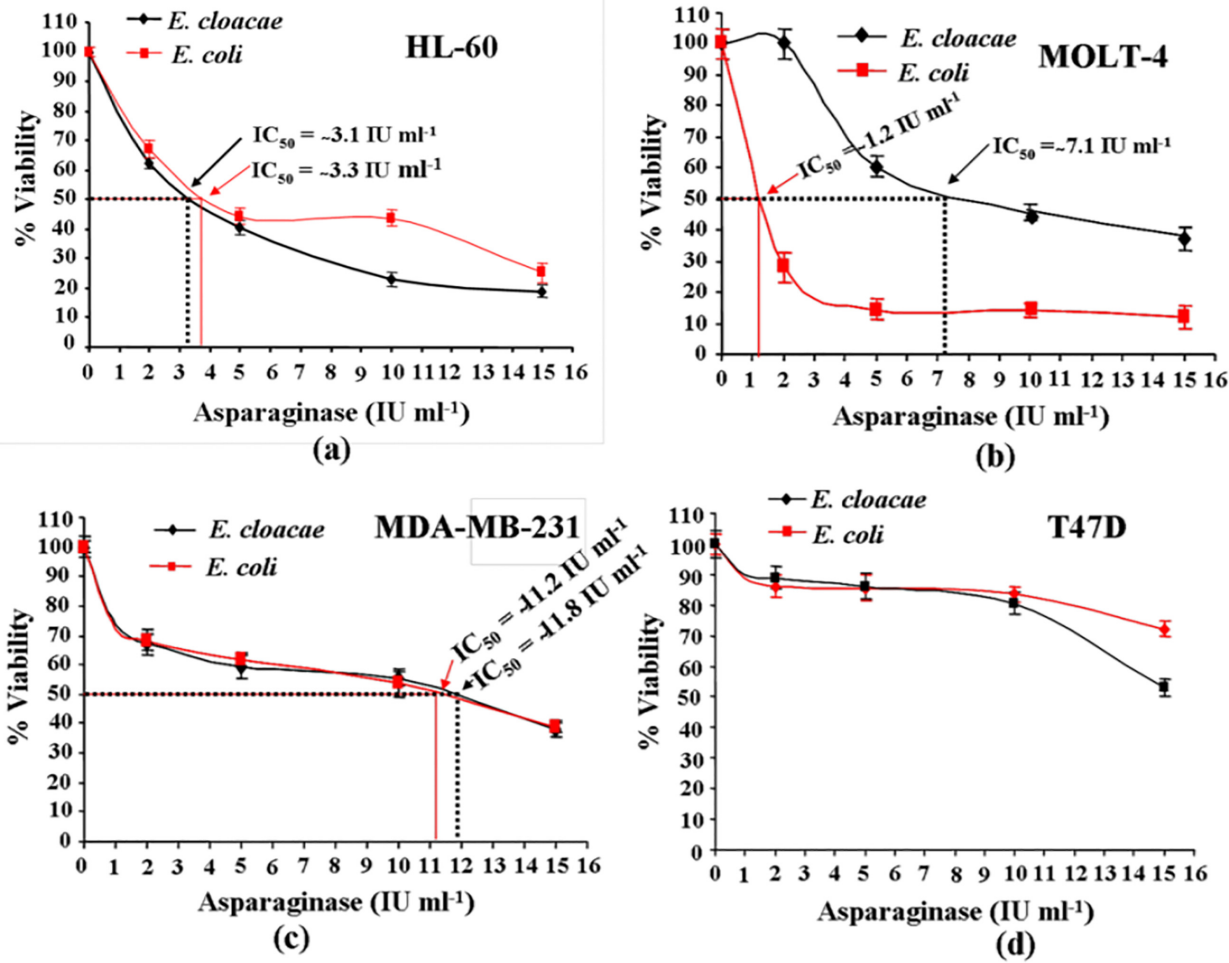
Dose-dependent effect of asparaginases on viability of human cancer cells. **(a-d)** MTT assay showing cytotoxic effect on HL-60, MOLT-4, MDA-MB-231, and T47D cells proliferation following treatment with purified asparaginase. Approximately, 1.5x10^4^ cells ml^-1^ were cultured at 37°C, separately. Purified *E*. *cloacae* asparaginase and reference asparaginase preparation (*E*. *coli* asparaginase) procured from Sigma chemical Co. St. Louis were added at a concentration of 2, 5, 10, and 15 IU ml^-1^. After 48 h of treatments cells viability was determined by the MTT assay. Each value represents the mean ±SD for three determinations.

We have used MTT assay to determine the cytotoxic effect of purified *E*. *cloacae* asparaginase on human leukemic cells HL-60, MOLT-4, and human breast cancer cells MDA-MB-231 and T47D. Cells growing in log phase were treated with 2, 5, 10, and 15 IU ml^-1^ concentration of asparaginase upto 48 h and results were represented in terms of half maximal inhibitory concentration (IC_50_). The standard *E*. *coli* asparaginase procured from Sigma chemical Co. St. Louis, USA, was used as a reference enzyme preparation. Results showed that asparaginase purified from *E*. *cloacae* was potently and significantly inhibit the proliferation of HL-60, MOLT-4 and MDA-MB-231 cells with IC_50_ values approximately 3.1, 7.1 and 11.8 IU ml^-1^, respectively. While, *E*. *coli* asparaginase exhibited IC_50_ values approximately 3.3, 1.2, and 11.2 IU ml^-1^ against HL-60, MOLT-4, and MDA-MB-231 cells, respectively. These results suggested that asparaginase affects viability of cells in a dose-dependent manner. However, IC_50_ values against T47D cells did not achieve till 15 IU ml^-1^ concentration of *E*. *cloacae* as well as with standard *E*. *coli* asparaginase. These observations suggest that the effect of asparaginase differs between cell types (Fig 5).

### Assessment of Cell Morphology

**Fig 6:**
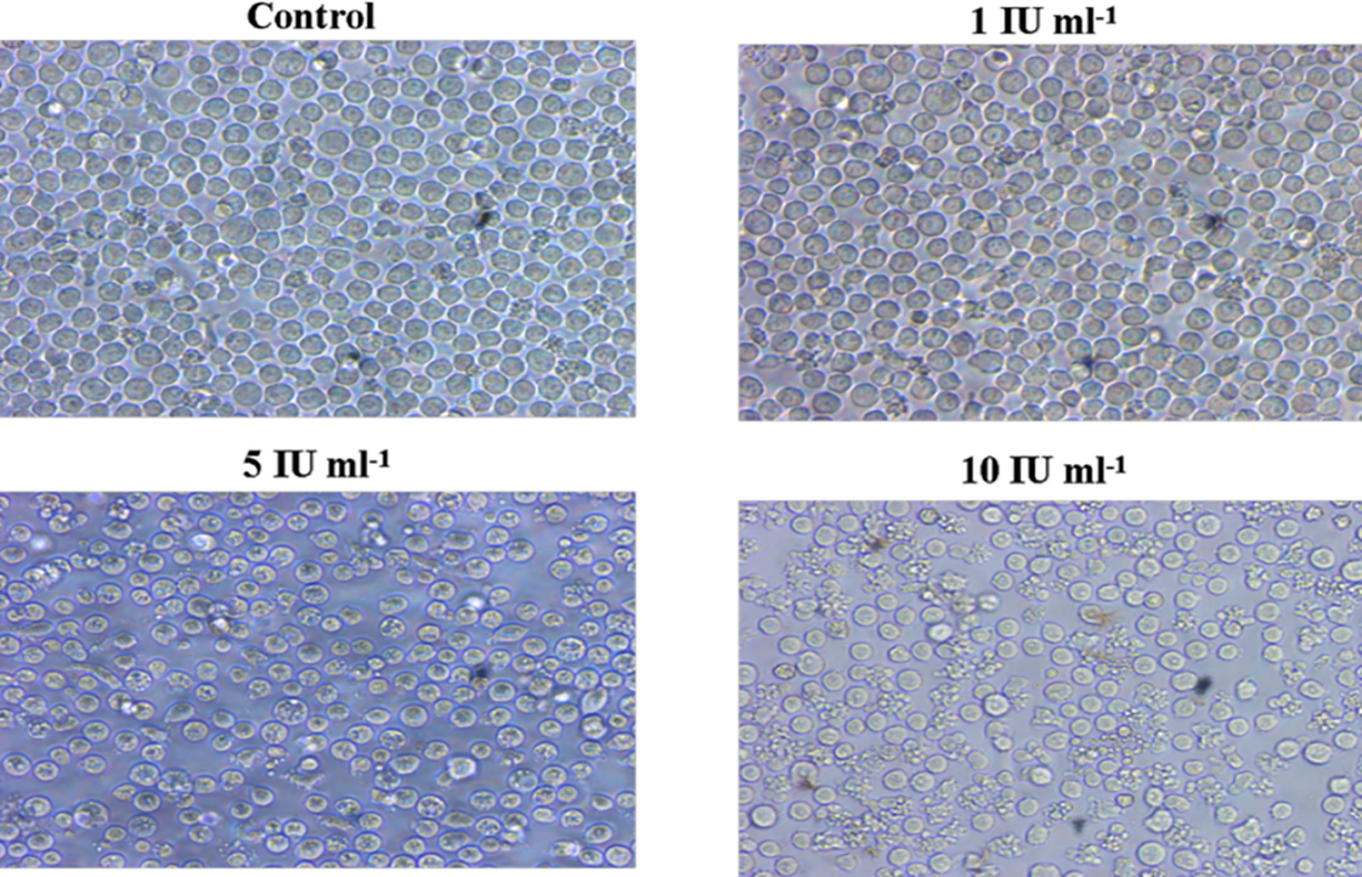
Asparaginase induced morphological changes on HL-60 cells. Cells were treated with the indicated concentrations of *E*. *cloacae* asparaginase and observed for morphological changes under microscope (1×81, Olympus). Photographs were taken by using DP-12 camera. The procedure is discussed in Materials and Methods.

The cytotoxicity induced by purified asparaginase was further confirmed by phase contrast microscopy. For this, HL-60 cells were treated with 1, 5, and 10 IU ml^-1^ of purified enzyme, after 24 h of treatment cells were subjected to investigation for morphological changes. Results showed that morphological changes were increased with increasing dose of asparaginase. At the concentration of 1 IU ml^-1^ in HL-60 cells, symptoms of apoptosis such as membrane blebbing and cell shrinkage were observed. The amount of blebbing and shrinkage of the cells were found to be increased dramatically at 5 and 10 IU ml^-1^ of enzyme treatment. However, morphological changes were not observed with untreated cells (Fig 6). These results further suggest that purified asparaginase induced potential apoptotic effect in dose-dependent manner.

### DAPI Staining for Assessment of Nuclear Morphology

**Fig 7:**
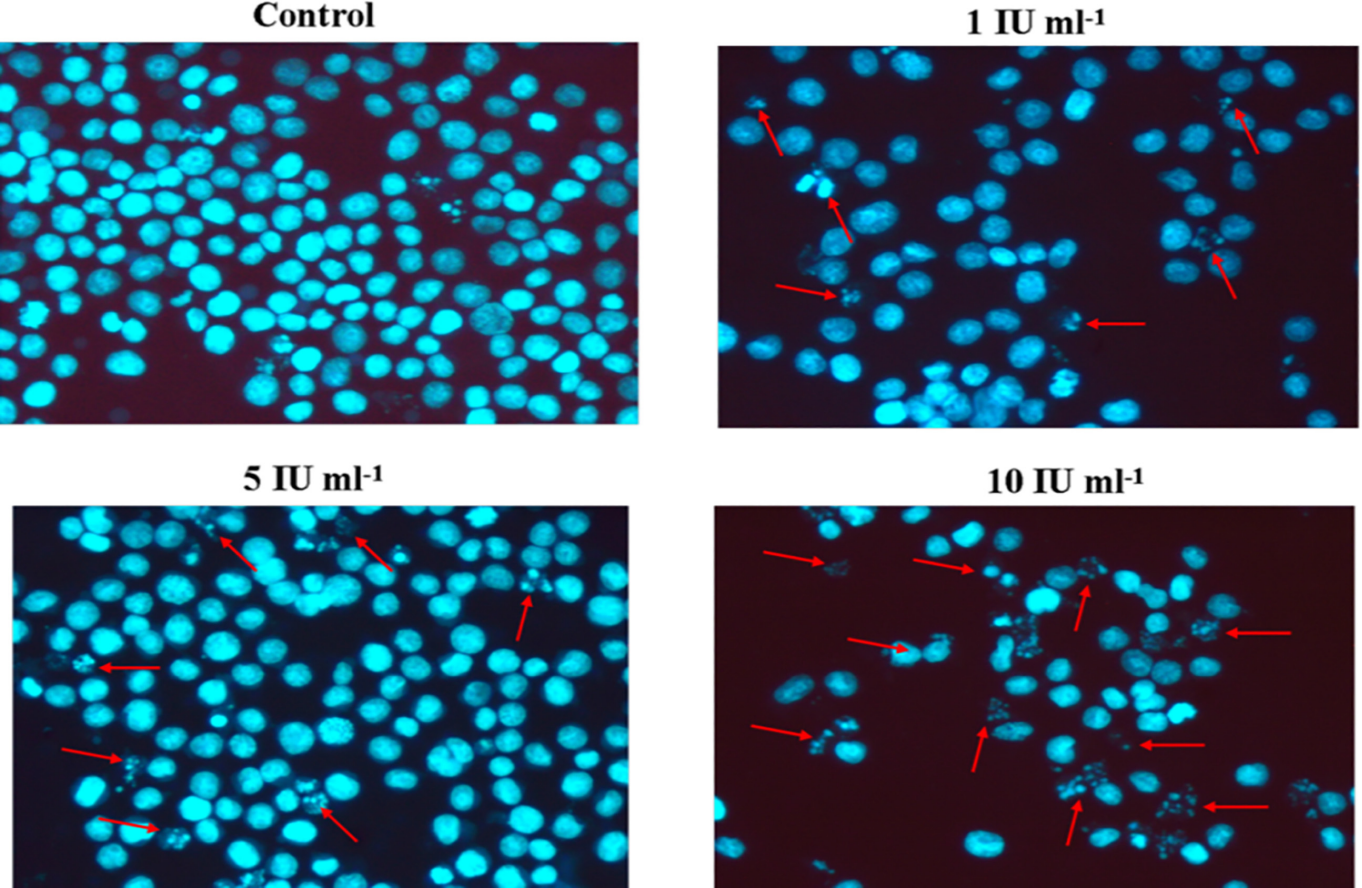
Alteration in nuclear morphology by treatment with purified *E*. *cloacae* asparaginase. HL-60 cells were treated with different concentrations of *E*. *cloacae* asparaginase, collected after centrifugation at 1600 rpm, washed once with PBS, and then stained with DAPI for 10 min. The procedure is discussed in Materials and Methods.

To investigate nuclear changes induced by *E*. *cloacae* asparaginase, HL-60 cells were treated with 1, 5, and 10 IU ml^-1^ enzyme for 24 h and stained with nuclear staining dye (DAPI). As displayed in Fig 7, cells undergoing asparaginase treatment clearly showed nuclear morphological sign of apoptosis such as shrinkage, chromatin condensation, naked DNA, DNA fragmentation and loss of normal nuclear architecture. However, bluish intact nuclei in control (untreated) cells indicated no alteration in nuclear morphology, suggesting that purified asparaginase induced alteration in nuclear morphology, which leads to apoptotic cell death of human leukemic cells.

### Asparaginase Induced G0/G1 Phase Cell Cycle Arrest in HL-60 Cells

**Fig 8:**
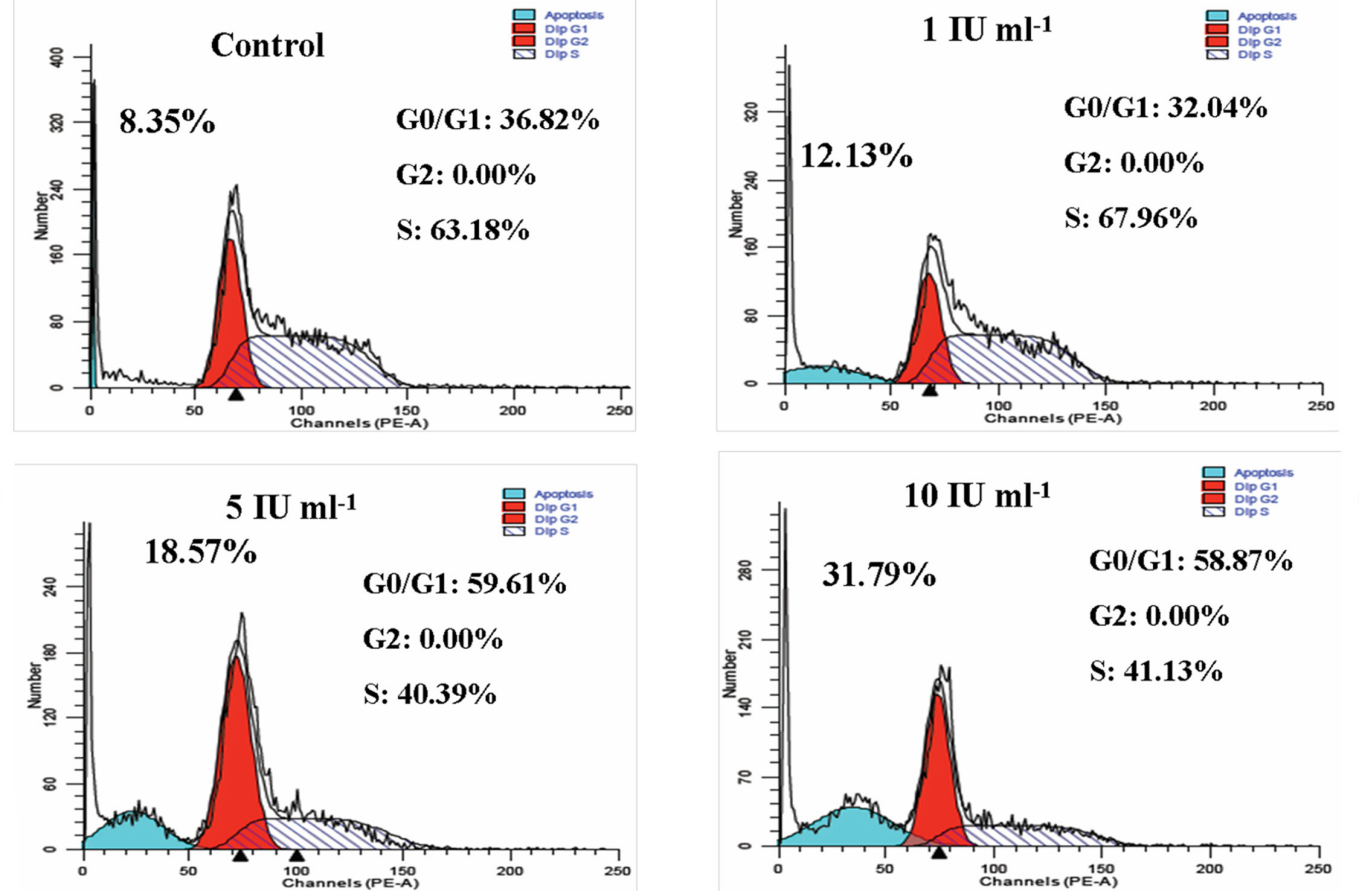
Effect of asparaginase purified from *E*. *cloacae* on cell cycle progression. HL-60 cells (1×10^6^) were seeded in 12-well plates and treated with different concentrations of purified *E*. *cloacae* asparaginase for 24 h. After treatment, cells were collected at 1600 rpm, washed once with PBS and fixed in 70% ethanol overnight. Cells were then washed once with PBS and stained with 100 μg of propidium iodide for 30 min. Modfit software was used to differentiate between phases and to determine the amount of apoptotic population. Histograms showed that G0/G1 phase arrest increases in dose dependent manner.

In order to determine whether the growth inhibition induced by purified asparaginase was due to replication defects, FACS analysis was performed. For this, we cultured HL-60 cells with different concentrations (1, 5, and 10 IU ml^-1^) of purified asparaginase. After treatment, cells stained with propidium iodide and subjected to flow cytometry. As summarized in Fig 8, the histogram of control cells (untreated) showed a standard cell cycle. Whereas, on treatment with 1, 5, 10 IU ml^-1^ of enzyme, cells showed 12.13%, 18.57%, and 31.79% apoptotic cell death, respectively. It is very interesting to note that at 1 IU ml^-1^ cells did not show any cell cycle arrest at G0/G1 phase. However, at 5 and 10 IU ml^-1^ treatment, cells showed almost equal cell cycle arrest at G0/G1 phase. Hence, these results suggest that proliferation inhibition could be mediated by a DNA replication defect followed by cell cycle arrest in G0/G1 phase.

### DNA Fragmentation by Agarose Gel Electrophoresis

**Fig 9:**
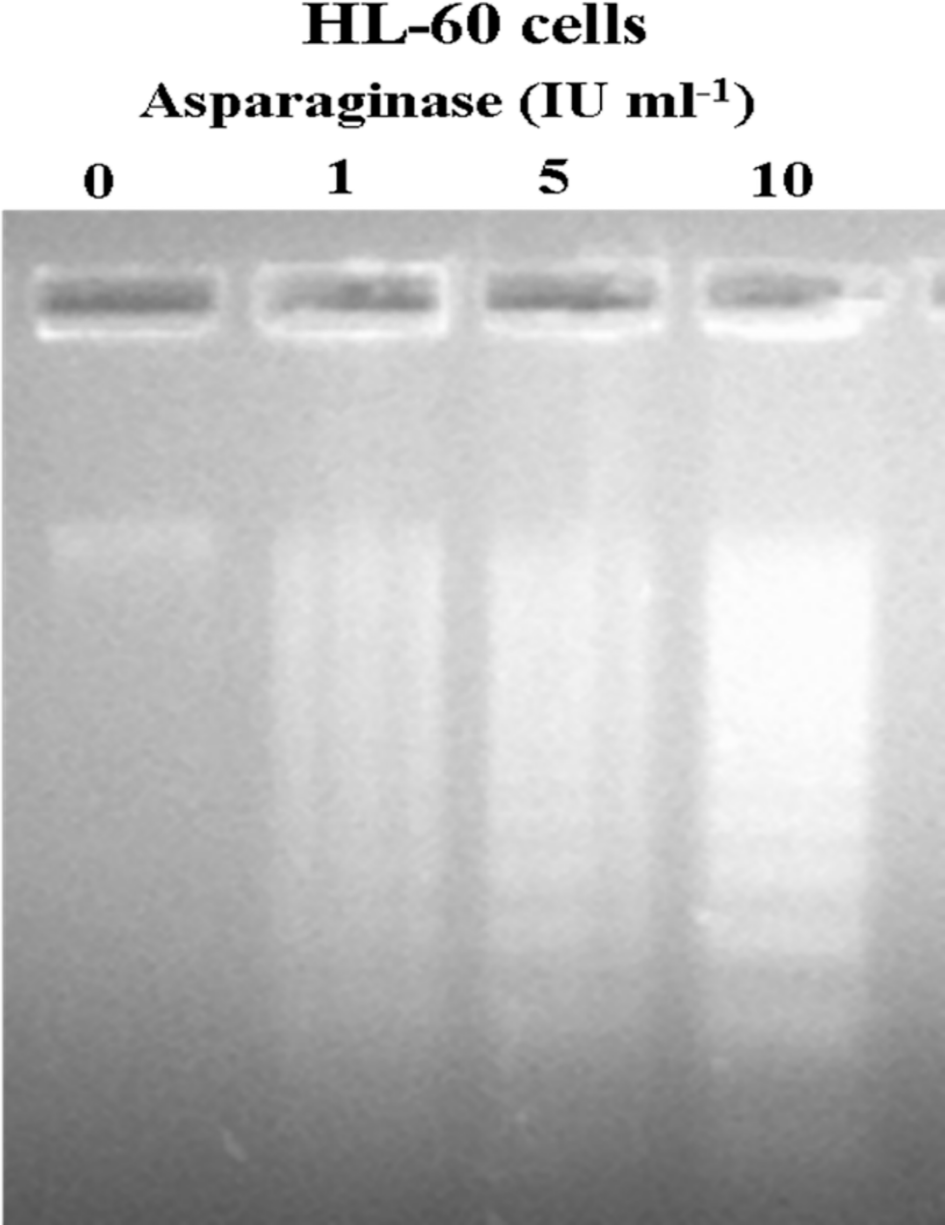
Agarose gel electrophoresis of genomic DNA extracted from HL-60 cells following the treatment of asparaginase. HL-60 cells treated with different concentrations of *E*. *cloacae* asparaginase for 24 h. Genomic DNA was isolated and electrophoresed on 1.8% agarose gel as described in Material and Method.

To investigate whether purified asparaginase treatment induced DNA fragmentation, HL-60 cells were treated with 1, 5, and 10 IU ml^-1^ enzyme for 24 h, genomic DNA was isolated and electrophoresed on agarose gel. According to the results that are presented in Fig 9, at low concentration, DNA ladder was barely visible and this became increasingly prominent in cells treated with higher concentration of asparaginase. However, DNA isolated from untreated cells did not show any DNA ladder. These results suggest that purified asparaginase severely damaged HL-60 cells by DNA fragmentation which leads to apoptosis.

### Acridine Orange-Ethidium Bromide (AO/EB) Staining for Detection of Apoptosis

**Fig 10:**
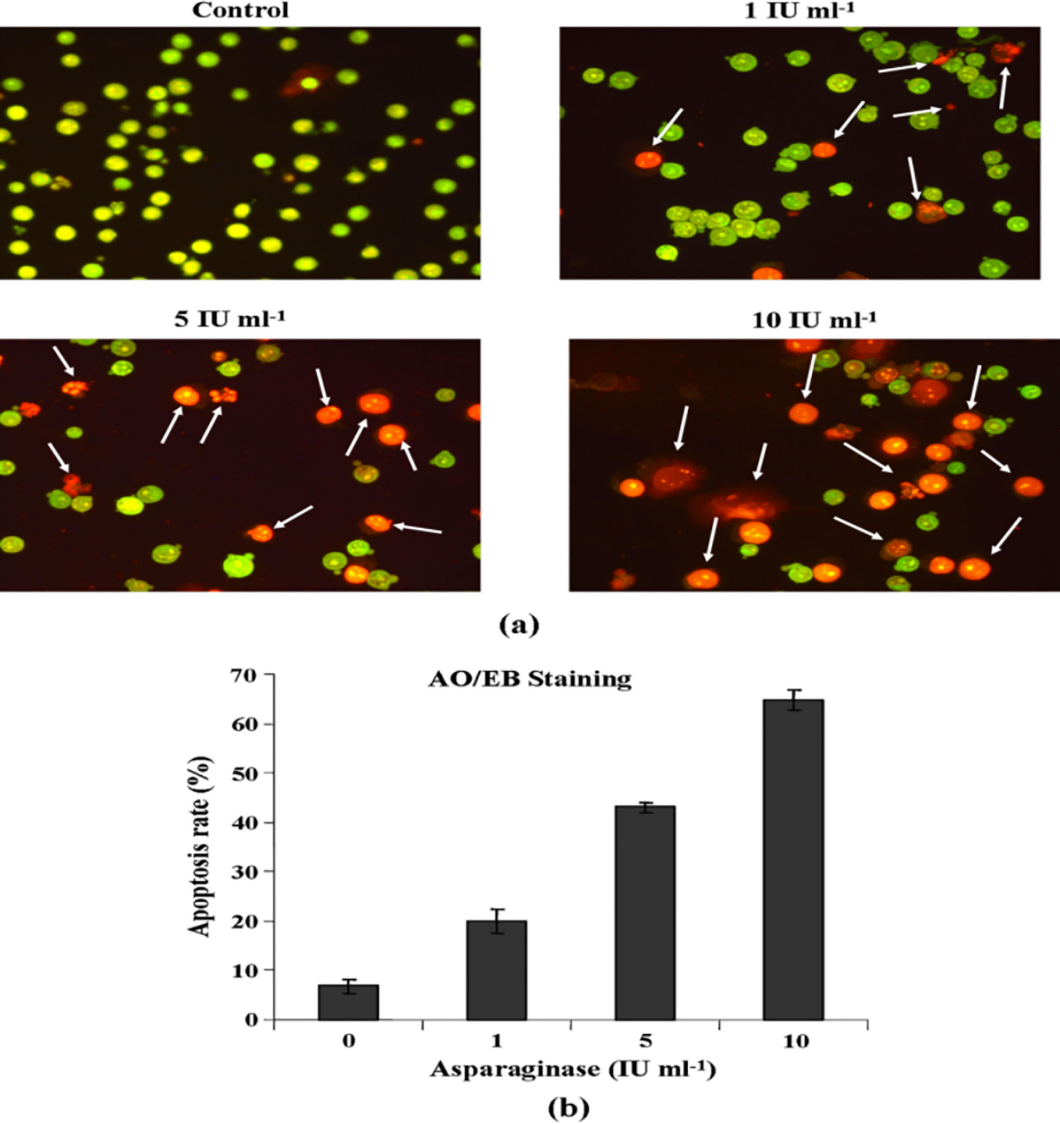
**(a) Morphological observation of asparaginase treated HL-60 cells after AO/EB double staining**. HL-60 cells treated with indicated concentrations of *E*. *cloacae* asparaginase for 24 h, stained with AO/EB solution and immediately visualized under a fluorescence microscope. Live cells showed green color nuclei and apoptotic cells showed orange color nuclei. Photographs were taken by using DP-12 camera. **(b)** Percentages of apoptosis were calculated during each concentration of asparaginase treatment. Values are means ±SD of three independent experiments.

Since, we observed accumulation of cells at G0/G1 peak during cell cycle analysis induced by asparaginase, further experiment was performed for quantification of apoptosis rate. For this, HL-60 cells were treated with 1, 5, and 10 IU ml^-1^ concentrations of asparaginase for 24 h, stained with AO/EB and microscopic examination was performed. As the results presented in Fig 10A showed that live cells in untreated control group displayed normal green color nuclei however, apoptotic cells showed orange color nuclei, which increases in dose dependent manner. The percent apoptosis rates, 6.89 ±1.73, 19.98 ±2.41, 43.02±1.07, and 64.63±2.11 were recorded at 0, 1, 5, and 10 IU ml^-1^ treatment of enzyme, respectively (Fig 10B). Hence, these results are corroborates with cell cycle analysis and supporting its use as an apoptosis inducing agent.

### Mitochondrial Membrane Potential (MMP) Loss or Δψm

**Fig 11:**
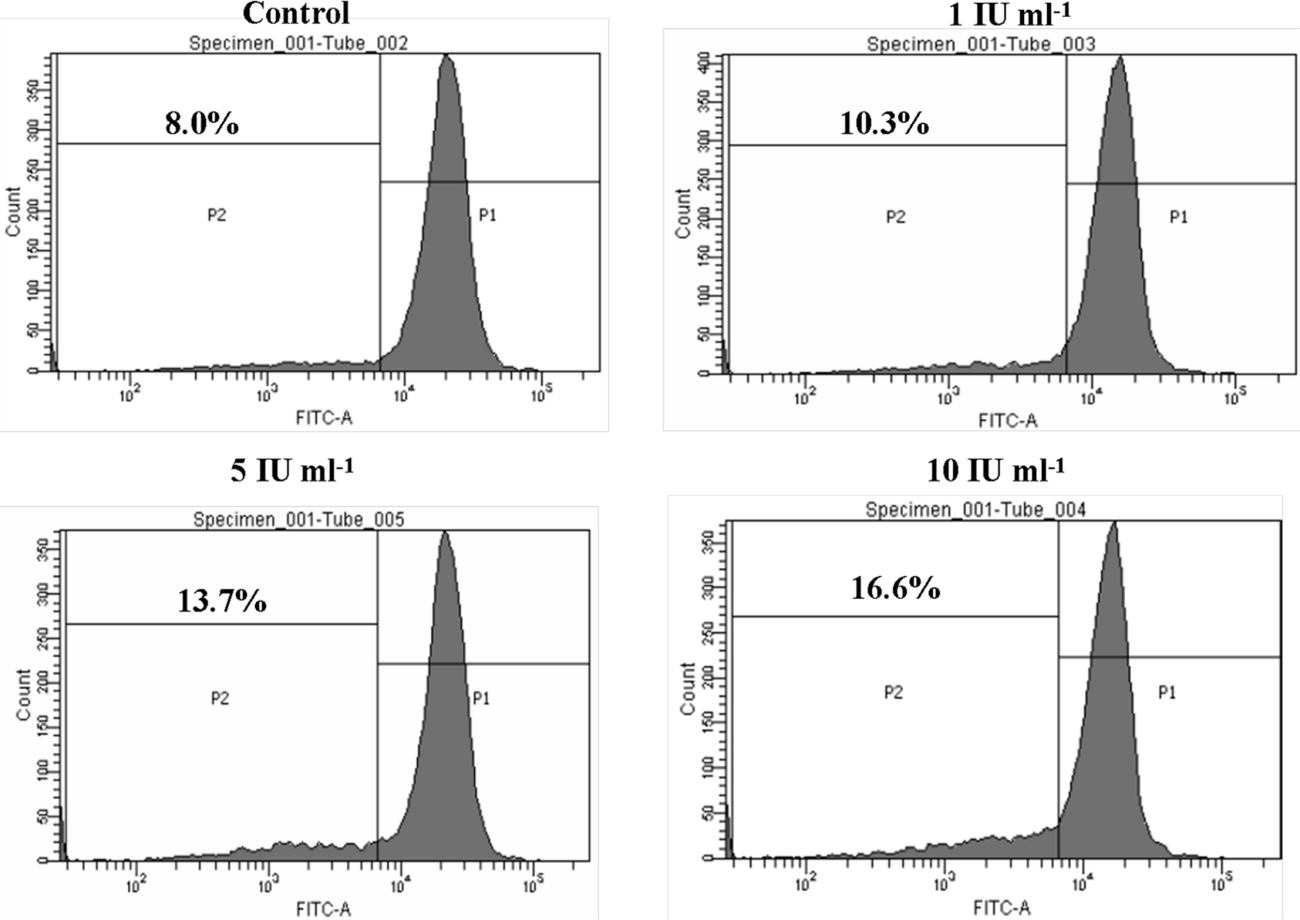
Asparaginase induced concentration dependent MMP loss in HL-60 cells. HL-60 cells (0.5×10^6^) were treated for 24 h with the indicated concentrations of purified *E*. *cloacae* asparaginase, washed once with PBS, and stained with Rhodamine-123. MMP was measured as discussed in Materials and Methods.

Mitochondrial integrity is required for cells to be functional and mitochondrial membrane potential loss is major characteristics of apoptosis. In order to explore the effect of *E*. *cloacae* asparaginase on mitochondrial membrane potential, HL-60 cells were cultured in presence of increasing concentration of purified enzyme, harvested after 24 h of treatment and subjected to flow cytometry analysis following the staining with Rhodamine-123. As results are summarized in Fig 11, 10.3%, 13.7%, and 16.6% cells showed mitochondrial dysfunctioning at 1, 5, and 10 IU ml^-1^ concentration of enzyme respectively, indicates that asparaginase disrupted the mitochondrial membrane and induces the apoptosis probably via activation of intrinsic pathway of cell death.

### Cytotoxic Effect on Normal Cells

**Fig 12:**
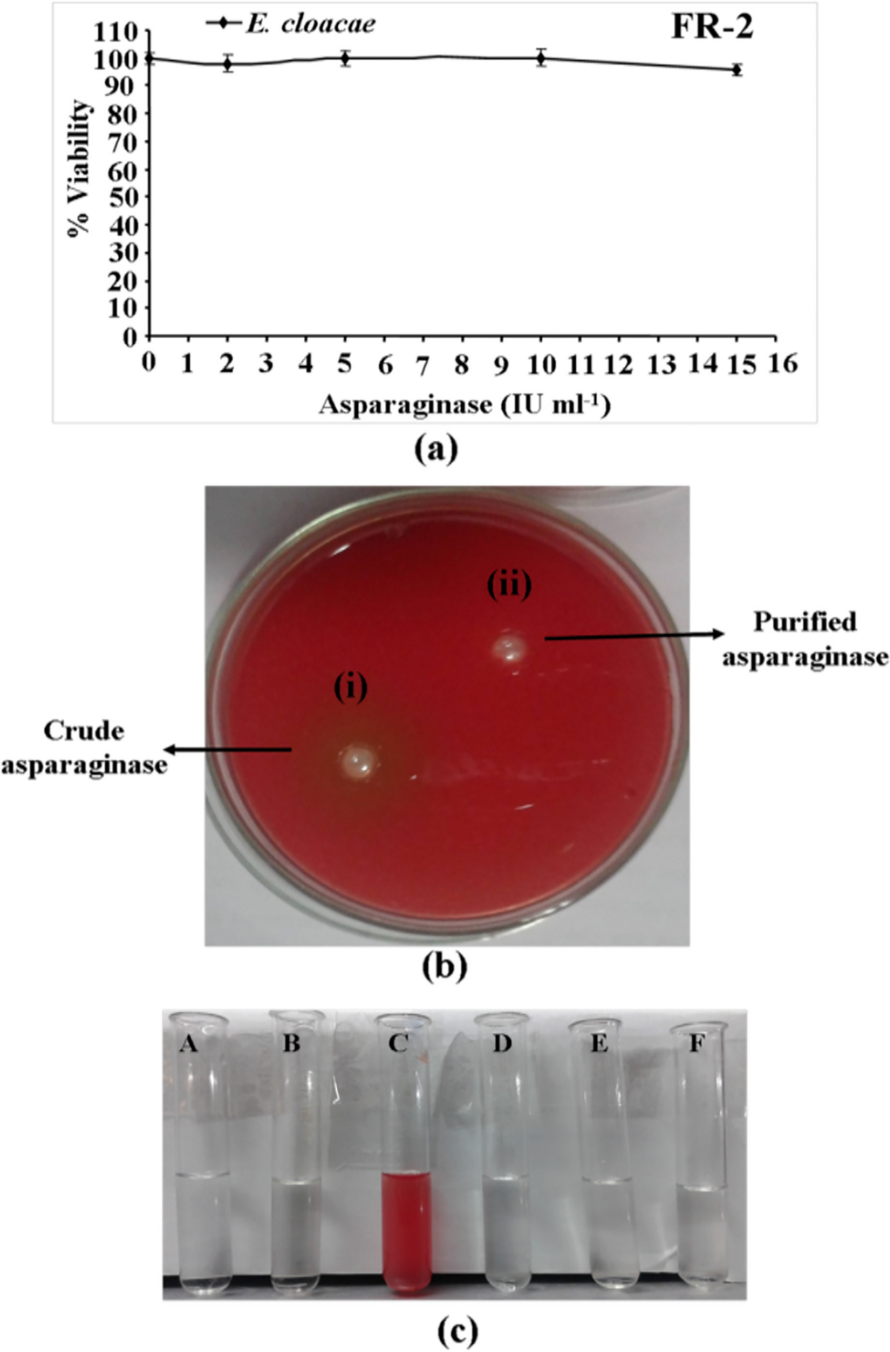
Toxicological evaluation of asparaginase purified from *E*. *cloacae*. **(a)** Non-toxic effects of purified asparaginase on noncancerous human epithelial cell line FR-2. Approximately, 1.5x10^4^ cells ml^-1^ were cultured at 37°C and treated with 2, 5, 10, and 15 IU ml^-1^ concentrations of purified asparaginasefor 48 h and cell viability was determined by the MTT assay. **(b(i)** Crude asparaginase formed clear translucent zone on blood agar plate due to lysis of erythrocytes. **(b(ii)** Purified asparaginase had no hemolytic effect. **(c)** Quantitative measure of the haemolytic activity. A: negative control (without asparaginase); B: positive control (Phosphate buffer); C: crude asparaginase; D: 15 IU ml^-1^; E: 7.5 IU ml^-1^; F: 3.75 IU ml^-1^ concentrations of asparaginase.

Since, MTT assay results showed that purified asparaginase induced proliferation inhibition in human leukemic as well as human breast cancer cells, we were interested to test its toxicity on normal cells. To study this, we cultured non-cancerous breast epithelial cells FR-2 and treated with 2, 5, 10 and 15 IU ml^-1^ concentrations of enzyme. Cells were harvested after 48 h and subjected to MTT assay. Results showed that purified enzyme did not affect cell viability upto 15 IU ml^-1^ concentration of enzyme (Fig 12A). These results suggesting that asparaginase purified from *E*. *cloacae* induced cytotoxicity on the tested human cancer cells and non-toxic for normal cells.

### Hemolysis Assays

The effect of the crude and purified asparaginase on human blood (erythrocytes) was investigated so as to determine whether the purified enzyme cause hemolysis. As shown in Fig 12B, crude enzyme formed a translucent zone on the blood agar plate, while hemolysis was not observed with purified enzyme. The results of quantitative hemolytic assay also showed that purified enzyme had no haemolytic effects till at highest concentration (15 IU ml^-1^) used in assay (Fig 12C). These results suggest that asparaginase purified from *E*. *cloacae* was non-toxic for erythrocytes.

## Discussion

Asparaginase is a well known chemotherapeutic enzyme, universally used in the treatment protocols of various neoplasms, particularly of lymphoid origins [30]. The commercially available asparaginases possesses 3–10% intrinsic glutaminase activity [20], which often produces several side effects including pancreatitis, hyperglycemia, neurological seizures and various hypersensitivity reactions [31]. Therefore, the search of glutaminase free asparaginase with effective antineoplastic activity has gained significant interest of modern researchers. In the current study, we report purification, characterization and evaluation of anticancer activity of a glutaminase free asparaginase purified from culture supernatant of *E*. *cloacae*. The enzyme was purified upto 119.39 fold which is comparable to the purity of therapeutic protein. The purified asparaginase has molecular weight approximately 106 kDa, assessed by Native-PAGE. While, subunit molecular weight was approximately 52 kDa assessed by SDS-PAGE and 2D-PAGE, suggests that purified enzyme was dimer of two identical molecular weight subunits. The difference in molecular weight as determined by SDS-PAGE and native-PAGE may be either due to the destruction of disulphide linkages or mass lost during boiling before loading protein sample onto SDS-PAGE. Above mentioned findings are in accordance with the previous observations stating that the molecular size of the bacterial origin asparaginases vary from 34 to 200 kDa [20,21, 32–36] and almost similar to the asparaginase purified from *E*. *aerogenes* (>100 kDa) [37]. The isoelectric point (*p*I) of purified enzyme was 4.5, which is slightly lower than the *p*I of asparaginase purified from *E*. *aerogenes* (5.3) [37] but closer to the *E*. *coli* asparaginase (5.0) [38]. Purified asparaginase from *E*. *cloacae* remained active and stable over wide range of pH and temperature and optimum activity showed between pH 7–8 and temperature 35–40°C, which is comparable to the pH of human circulating system and body temperature. These results suggest that purified asparaginase can adopt rapidly and function normally in internal environment of human body. The monovalent cations, Na^+^ and K^+^ were found to enhanced asparaginase activity, indicates that the enzyme might contain Na^+^ and K^+^ ions. While, no inhibition was observed when enzyme was incubated with EDTA, indicates that purified enzyme is not a metallo-enzyme. These results are in accordance with the previously purified asparaginases from *Bacillus licheniformis* [36] and *Pectobacterium carotovorum* MTCC1428 [20]. Reducing agents such as 2-mercaptoethanol, glutathione (reduced) were found to enhanced enzyme activity while, thiol group blocking agent such as iodoacetamide and metal ions viz. Cd^2+^, Ni^2+^, and Hg^2+^ were found to inhibit the enzyme activity, indicates the presence of free sulfhydryl (-SH) groups at the active sites of enzyme. Purified enzyme hydrolyzed its natural substrate L-asparagine and did not hydrolyze D- or L-glutamine, L-glutamic acid and urea etc. which can be medically more important. The *K*_*m*_ of purified asparaginase was 1.58×10^−3^ M, which is lower than asparaginases purified from *Erwinia aroideae* (3×10^−3^ M) [39], *E*. *coli* (3.5×10^−3^ M) [40], *Corynebacterium glutamicum* (2.5×10^−3^ M) [41], *Tetrahymena pyriformis* (2.2×10^−3^ M) [42], *Thermus thermophilus* (2.8×10^−3^ M) [33], and *Bacillus subtilis* (2.06×10^−3^ M) [43], suggests that asparaginase purified from *P*. *otitidis* possess high substrate affinity towards its natural substrate asparagine.

Serum [44] and pancreatic enzymes such as trypsin [45] are the major barriers that inactivate/hydrolyze clinically administrated asparaginase, resulting in reduction of asparaginase bioavailability [9]. The *in vitro* serum and trypsin half life of purified asparaginase was ~ 39 h and ~ 32 min respectively, which is higher than previously reported *in vitro* serum (20 h) [10] and trypsin (26 min) [46] half life of commercial *E*. *coli* asparaginase (native). These observations collectively suggest that purified asparaginase could be considered as a drug with longer plasmatic half life. Asparaginases have been reported from various sources but all asparaginases do not possess cytotoxicity [47,48]. Hence, the cytotoxicity of asparaginase purified from *E*. *cloacae* was tested against panel of human cancer cell lines, HL-60, MOLT-4, MDA-MB-231 and T47D. The cytotoxic efficacy of purified asparaginase against HL-60, MDA-MB-231 and T47D cells is comparable to commercial *E*. *coli* asparaginase (sigma). However, against MOLT-4 cells commercial asparaginase showed better cytotoxicity. These results indicate that purified asparaginase induces apoptosis with different kinetics in tested human cancer cell lines. The highest cytotoxicity of the enzyme was observed against human promyelocytic leukemia cells HL-60. Hence, further studies were carried out with this cell line. The morphological changes during apoptosis include membrane blebbing, cell shrinkage, chromatin condensation, formation of apoptotic and scattered apoptotic bodies. By performing phase contrast and fluorescence microscopy, we noted that after 24 h of treatment with *E*. *cloacae* asparaginase, HL-60 cells showed all aforementioned morphological changes, which were increased in dose-dependent manner. Similar observations were also recorded with asparaginase treated mouse lymphoma [49] and human T lymphocytes cells [50] and with other molecules recently tested as potent antileukemic agent [51, 52].

Cell cycle deregulation, specifically at cell-cycle check points have previously been highlighted as a common modality that leads to cancer. By targeting these check points with various compounds may lead to development of effective anticancer drugs [53]. Hence, we examined the cell cycle profile by FACS analysis. The data of cell cycle phase distribution analysis showed a significant accumulation of cells in the G0/G1 phase which consist of cells either apoptotic or necrotic population. The authors suggest that G0/G1 phase arrest might be associated with down-regulation of cell cycle regulator proteins, cdc2, cdk2, and cdk3 and up-regulation of tumor suppressor protein such as p53. Interestingly, it was noted that S phase was decreased dramatically at 5 and 10 IU ml^-1^ of enzyme treatment, which needs further investigation. These observations revealed that purified asparaginase from *E*. *cloacae* induced apoptosis by arresting cell cycle in G0/G1 phase. Analysis of fragmented DNA from apoptotic cells by agarose gel electrophoresis is known to produce a characteristic DNA ladder that is widely regarded as a biochemical hallmark of apoptosis [26]. Hence, we attempted to investigate the ability of asparaginase to induce DNA damage of the cells. For this, we performed DNA fragmentation assay which showed DNA damage and fragmentation occurring in genome. These observations indeed suggest that purified asparaginase triggers apoptosis through DNA damage. Further, in order to delineate the apoptotic potential of purified asparaginase, we attempted AO/EB staining and it was observed that the rate of apoptosis (orange color nuclei) was increased in dose dependent manner.

Mitochondrial membrane potential (MMP or Δψm) loss is an essential event of apoptosis [54] and the measurement of MMP loss is one of the methods used for investigation of apoptosis [51]. Hence, further we attempted to understand the effect of purified enzyme on mitochondrial membrane permeability in HL-60 cells. We noted a limited increase in MMP loss after 24 h of treatment. We suggest that the MMP loss induced by purified enzyme might be via activation of proapoptotic proteins and down-regulation of antiapoptotic proteins involved in intrinsic pathway of cell death, but further studies are required to understand the pathway of apoptotic cell death of tested human leukemic HL-60 cells. Chemotherapy is one of the methods used for the treatment of neoplasm [55]. Today several drugs are used in cancer chemotherapy and many more are under clinical trials but they often have poor selectivity and cause toxicity on normal cells [56,57]. The purified asparaginase from *E*. *cloacae* did not inhibit the proliferation of noncancerous FR-2 cells, upto 15 IU ml^-1^. While, at this concentration >80% cells viability of HL-60 cells was reduced. This observation suggests that purified enzyme selectively suppresses the proliferation of cancer cells and nontoxic for normal cells. Hemolysis is one of the main problem encountered with protein drugs [11] and compounds from biological sources which are nontoxic or expected to have least toxic effects on blood cells, is the main focus of cancer therapy today [58]. By performing the *in vitro* hemolysis assays, we noted that purified enzyme had no hemolytic effect on human blood cells. These observations suggest that purified asparaginase is hemocompatible and could be safe to use as a chemotherapeutic agent.

In conclusion, a glutaminase free asparaginase was purified to electrophoretic homogeneity from indigenous bacterium *E*. *cloacae*. The biochemical and physiological characteristics revealed that purified enzyme easily adopt or act more effectively in internal environment of human body. The cytotoxic potential of purified asparaginase is comparable to commercial asparaginase. Mechanistically, purified enzyme induced apoptosis by arresting cell cycle in G0/G1 phase and also dysfunctioning of mitochondrial integrity. It is noteworthy that *E*. *cloacae* asparaginase is nontoxic for human noncancerous (FR-2) cells and had no hemolytic effect on erythrocytes.
